# Poly(ADP-ribose) polymerase-1 gene in human tumor cell lines: Its expression and structural alteration

**Published:** 2004-02-01

**Authors:** Mitsuko Masutani, Tadashige Nozaki, Hiroki Sasaki, Tesshi Yamada, Takashi Kohno, Kimiko Shimizu, Masahiro Gotoh, Masahiko Shiraishi, Jun Yokota, Setsuo Hirohashi, Hitoshi Nakagama, Takashi Sugimura

**Affiliations:** *1) Biochemistry Division, National Cancer Center Research Institute, 5-1-1, Tsukiji, Chuo-ku, Tokyo 104-0045, Japan; *2) Genetics Division, National Cancer Center Research Institute, 5-1-1, Tsukiji, Chuo-ku, Tokyo 104-0045, Japan; *3) Experimental Pathology and Chemotherapy Division, National Cancer Center Research Institute, 5-1-1, Tsukiji, Chuo-ku, Tokyo 104-0045, Japan; *4) Biology Division, National Cancer Center Research Institute, 5-1-1, Tsukiji, Chuo-ku, Tokyo 104-0045, Japan; *5) Molecular Oncology Division, National Cancer Center Research Institute, 5-1-1, Tsukiji, Chuo-ku, Tokyo 104-0045, Japan; *6) Pathology Division, National Cancer Center Research Institute, 5-1-1, Tsukiji, Chuo-ku, Tokyo 104-0045, Japan; *7) DNA Methylation and Genome Function Project, National Cancer Center Research Institute, 5-1-1, Tsukiji, Chuo-ku, Tokyo 104-0045, Japan

**Keywords:** Poly(ADP-ribose) polymerase-1, tumor cell line, gene expression, structural alteration

## Abstract

Poly(ADP-ribose) polymerase-1 (Parp-1) is involved in DNA repair and cell-death induction after DNA damage. *Parp-1^−/−^* mice show higher susceptibility to the carcinogenic effects of nitrosamine and azoxymethane. To elucidate the role of alterations of the *PARP-1* gene in human carcinogenesis, we examined the expression level of *PARP-1* gene in various human tumor cell lines. The presence of gross rearrangement of *PARP-1* gene in these cell lines was also examined by Southern blot hybridization analysis. The expression levels of *PARP-1* gene in several cell lines, including T-cell leukemia cell lines (Molt-4 and CCRF-CEM), colon cancer cell line (WiDr), and gastric cancer cell lines (KATOIII, OKAJIMA, and MKN45) was substantially lower than in other cancer cell lines. Among the 85 analyzed cell lines, structural alteration of *PARP-1* gene was detected in a gastric cancer cell line, MKN28. A low level of PARP-1 expression in human cancer could potentially influence cancer cell growth, differentiation and cancer development by affecting genomic instability, as well as the response of tumors to chemo- and radiotherapy.

## Introduction

Poly(ADP-ribose) polymerase-1 (PARP-1) catalyzes polyADP-ribosylation of proteins using NAD as a substrate after activation by DNA strand breaks.1)–3) PARP-1 has a regulatory role in base excision repair (BER) and DNA strand break repair by interacting with proteins involved in these repair pathways and modifying them.4) In *Parp-1^−/−^* mice, a higher frequency of deletion mutations was observed than in *Parp-1^+/+^* mice after treatment with carcinogenic *N-*nitrosobis(2-hydroxypropyl)amine (BHP),5) suggesting that enhanced genomic instability contributes to higher susceptibility to the carcinogenic effects of BHP in *Parp-1* deficiency.6) PARP-1 is also activated by DNA damages, its activation induces depletion of cellular NAD level7) and consequently stimulates apoptosis-inducing factor-dependent cell death.8)

Although the evidence suggests that PARP-1 is likely to be involved in human carcinogenesis, alteration of the *PARP-1* gene in human cancer has not been fully studied yet, but several reports described the level of *PARP-1* gene expression9) and alteration of the structure of *PARP-1* gene in various malignancies.10)–12) For example, Bhatia *et al*.10)–12) reported that the allele frequency of a polymorphism in the processed pseudogene of *PARP-1* on chromosome 13q33-qter is increased in Burkitt lymphoma, multiple myeloma, colon and prostate cancers in African American populations. Furthermore, Bieche *et al*.13) reported that low *PARP-1* gene expression correlated with higher genomic instability in breast cancer. Prasad *et al*.9) reported that in comparison to other tumor cell lines, Ewing’s sarcoma cell lines exhibited a high constitutive level of PARP-1 activity and an increased level of *PARP-1* mRNA expression.

Since *Parp-1^−/−^* cells show enhanced sensitivity to alkylating agents and *γ*-irradiation,14)–16) the decreased level of PARP-1 in cancers might substantially affect the genomic stability of the cells as well as the responses to chemo- and radiotherapy. In this study, we examined the expression levels of *PARP-1* mRNA in various tumor cell lines and the presence of gross structural alterations in the *PARP-1* gene by Southern blot hybridization analysis.

## Materials and methods

### Cell lines

Studies were conducted using 11 colon cancer cell lines (HCT15, WiDr (HT29), SW1116, LMCO5, Colo201, Colo205, LMCo3, LMCO4, PMCO1, Colo320DMF, and Colo321), 8 liver cancer cell lines (HepG2, Alex, Li7NM, Li7HM, Li21, Li22, Li23, and Li24), 9 gastric cancer cell lines (MKN7, MKN1, MKN28, MKN74, TMK1, KATOIII, OKAJIMA, HSC39, and MKN45),17) 48 lung cancer cell lines (Ma-1, Ma-2, Ma-10, Ma-12, Ma-17, Ma-24, Ma-25, Ma-26, Ma-29, PC-13, Lu-99A, MS-18, H209, H774, H841, RERF-LC-MS, H23, H157, H332, H441, H520, H526, H596, H1155, Lu65, H82, H69, Lu24, Lu134,, N417, SBC-5, EBC-1, A427, A549, PC-1, PC-3, PC-7, PC-9, PC-10, LC1-Sq, RERF-LC-OK, VMRC-LCD, ABC-1, Ma-25, H774, H322, Lu99, and Lu65),18) 3 cervical cancer cell lines (Ncc-CX1, HeLa229, and SiHa),19) 5 renal cancer cell lines (RCC23, RC3, RC4, KPK1, and KN41), and 13 esophageal cancer cell lines (TE1, TE2, TE3, TE4, TE5, TE6, TE7, TE8, TE9, TE10, TE11, TE12, and TE13).17),20) We also used 2 osteosarcoma cell lines (Saos-2, and U2OS), 2 Ewing’s sarcoma cell lines (SK-ES-2, and RD-ES), 18 leukemia/lymphoma cell lines (Kasumi-1, Namalva, 697, GM607, Molt-4, HEL, CCRF-CEM, CMK, U937, THP-1, Takeda, K562, HL60, S.S., J111, T-ALL, Kawai, and SCC-3).21) Three surgical specimens of human lung cancer were also examined.22)

### Northern and Southern blot analysis

Northern and Southern blot analyses were performed as described previously.17) As a probe, we used a 3.0 kb cDNA of *PARP-1*, encompassing the entire coding region of the *PARP-1* gene.23)

### Measurement of PARP-1 activity

PARP-1 activity was measured by incorporation of radioactivity derived from ^32^P-NAD (New England Nuclear, Beverly, MA) into an acid-insoluble fraction as described previously.24) Briefly, crude cell extract was incubated with an assay mixture containing 50 mM Tris-HCl (pH8.0), 10 mM MgCl_2_, 1 mM dithiothreitol, 2 μg/ml activated DNA (Sigma Chemical Co., St. Louis, MO), 2 μg/ml calf thymus histone (Sigma), and 100 μM ^32^P-NAD (8 Ci/mmol) at 25 °C for 10 min. The reaction was stopped by addition of trichloroacetic acid to 5% and the radioactivity in precipitate was measured after filtration through glass-filter (Whatman, Clifton, NJ). One unit of PARP activity was defined as an incorporation of 1 pmol of NAD per min at 25 °C.

## Results and discussion

**Fig. 1:**
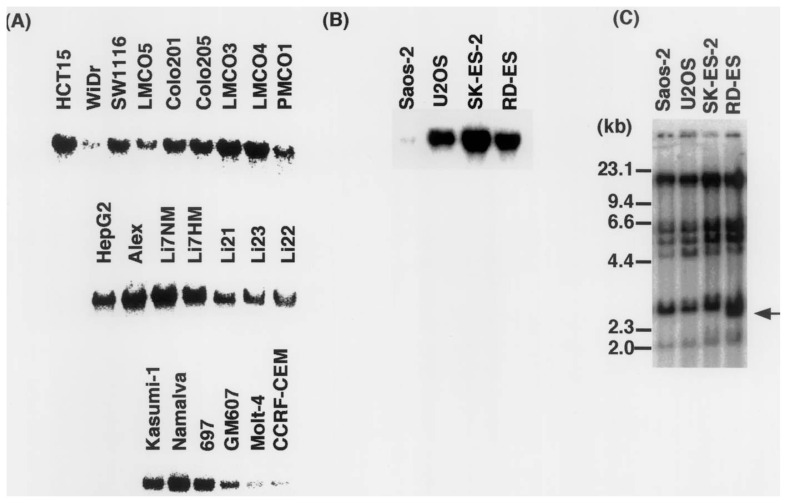
Downregulation of *PARP-1* expression in tumor cell lines detected by northern blot analysis and Southern blot analysis of the *PARP-1* gene in sarcoma cell lines. (A) Upper, middle and lower panels show colon and liver cancer and leukemia cell lines, respectively. (B) Oseteosarcoma cell lines (Saos2 and U2OS) and Ewing’s sarcoma cell lines (SK-ES-2 and RD-ES).2) Southern blot analysis of the *PARP-1* gene after digestion of genomic DNA with *Hin*dIII. Arrow indicates the extra *PARP-1* gene fragment corresponding to a polymorphism in the pseudogene of *PARP-1* in RD-ES.

*PARP-1* mRNA expression was analyzed by northern blot analysis in 9 colon tumor cell lines, 7 liver cancer cell lines, 6 leukemia cell lines, and 4 sarcoma cell lines (Fig. 1A & B). Colon cancer cell line (WiDr),25) osteosarcoma cell line (Saos-2),9),26) and acute lymphoblastic T-cell leukemia cell lines (Molt-427) and CCRF-CEM28)) showed lower expression levels of *PARP-1* mRNA compared to other cell lines. Examination of osteosarcoma and Ewing’s sarcoma cell lines for the presence of gene rearrangement by Southern blot analysis found that the RD-ES cell line harbored an extra fragment of 2.6 kb after *Hin*dIII digestion of genomic DNA (Fig. 1C, arrow). Production of this fragment was due to a polymorphism in the processed pseudogene of *PARP-1* on chromosome 13q33-qter, as determined after digestion with genomic DNA with *Pst*I as reported by Bhatia *et al*.11) The 193-bp duplication within the A allele and its absence in the B allele is the source of polymorphism. The 2.6-kb *Hin*dIII fragment corresponded to the B allele. PCR analysis showed that RD-ES harbors both A and B alleles whereas Saos-2, U2OS, and SK-ES-2 contained only the A allele (data not shown). Previous studies reported the association of higher B allele frequency with endemic Burkitt lymphoma, multiple myeloma, colon and prostate cancers in African American populations.10)–12) Prasad *et al*.9) reported that the RD-ES cell line expresses a normal-size PARP-1 protein and shows a comparable level of basal PARP-1 activity. Further studies are necessary to clarify whether treatment of RD-ES cell line with DNA damaging agents results in altered level of PARP activity.

**Fig. 2:**
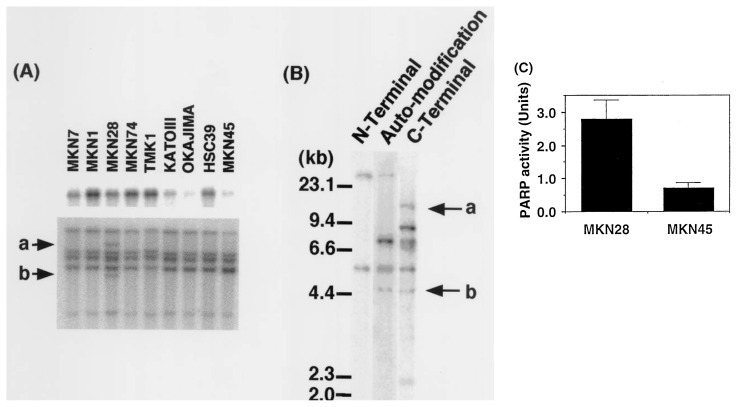
Downregulation of *PARP-1* expression in gastric cancer cell lines and rearrangement of the *PARP-1* gene in MKN28. (A) Upper panel: expression level of *PARP-1* mRNA detected by northern blot analysis. Lower panel: Southern blot analysis of the *PARP-1* gene after *Eco*RI digestion. Arrows “a” and “b” indicate extra fragments observed in MKN28. (B) Southern blot analysis of the *PARP-1* gene after *Eco*RI digestion using N-terminal, automodification and C-terminal probes of the *PARP-1* gene.2) PARP-1 activity was measured by incorporation of radioactivity derived from ^32^P-NAD into an acid-insoluble fraction. Data are mean ± SE.

Three gastric cancer cell lines, KATOIII, OKAJIMA and MKN45,29) also demonstrated low expression levels of *PARP-1* gene compared to six other gastric cancer cell lines (Fig. 2A, upper panel). The low level of *PARP-1* gene expression in these tumor cell lines could be due to either inactivation of *PARP-1* promotor by DNA hypermethylation or mutation, or by downregulation of some transcription factors involved in the regulation of *PARP-1* gene expression.

Furthermore, MKN2829) was found to harbor two extra fragments of *PARP-1* as indicated by Southern blot hybridization analysis after *Eco*RI digestion of genomic DNA (Fig. 2A, lower panel, Arrows “a” & “b”). We observed the presence of an extra fragment of *PARP-1* also after digestion with *Hin*dIII, *Bam*HI, and *X*baI (data not shown). These results suggest that MKN28 contains the structural alteration in the *PARP-1* gene. When the *PARP-1* cDNA probe, encompassing the entire coding region, was divided into N-terminal, automodification, and C-terminal domains, the extra fragment “a” hybridized only to the C-terminal probe, whereas the fragment “b” hybridized to both automodification and C-terminal probes. This suggests that the structural alteration spans the auto-modification to C-terminal domains of the *PARP-1* gene. The probe used for Southern blot hybridization analysis is a cDNA encompassing the whole coding region of the *PARP-1* gene and it detects *PARP-1* gene on chromosome 1q41-q42 and pseudogenes on chromosome 13q33-qter and on chromosome 14q22.11) Further studies are necessary to clarify whether the rearrangement also involves the pseudogenes. Little or no information is available on whether the rearrangement is continuous or not and whether it is caused by intragenic or intergenic recombination. Since the densities of fragments “a” and “b” were approximately half compared to other bands, the rearrangement was considered as a monoallelic event.

We measured PARP-1 activity using cell extracts of MKN28 and MKN45, as shown in Fig. 2C, and found that the former had a four-fold higher activity of PARP-1 than the latter. That the activity pattern correlated with *PARP-1* mRNA expression level implies that the structural alteration in MNK28 did not affect the basal activity of PARP-1. It is yet to be clarified whether the enzymatic activity or the function of PARP-1 under DNA damaging conditions is influenced by this structural alteration of the *PARP-1* gene in MKN28.

Gene rearrangement was further screened in 48 lung cancer cell lines (described in Materials and methods), 13 esophageal cancer cell lines (TE1, TE2, TE3, TE4, TE5, TE6, TE7, TE8, TE9, TE10, TE11, TE12, and TE13), 3 cervical cancer cell lines (Ncc-CX1, HeLa229, and SiHa), 3 colon cancer cell lines (Colo205, Colo320DMF and Colo321), 5 renal cancer cell lines (RCC23, RC3, ACHN, KPK1, and KN41) and 13 lymphoma/leukemia cell lines (Molt-4, HEL, CMK, U937, THP-1, Takeda, K562, HL60, S.S., J111, T-ALL, Kawai, and SCC-3), and 3 primary lung cancer tissues (data not shown). No rearrangement in the *PARP-1* gene was observed in these samples by Southern blot hybridization analysis with *Eco*RI digestion indicating that gross rearrangement in the *PARP-1* gene may be a rare event in cancer cells.

To determine the role of PARP-1 dysfunction in carcinogenesis, further analysis of *PARP-1* gene mutation and polymorphism should be conducted at the sequence level by refined methods, including PCR-SSCP (single-strand conformation polymorphism) and direct sequencing. Reduced polyADP-ribosylation activity was demonstrated in patients with Werner Syndrome after treatment with an alkylating agent, methylmethanesulfonate, but was not observed without any treatment or after treatment with bleomycin,30) indicating that aberration of PARP-1 activity should be examined after treatment with various types of DNA damaging agents as well.

In conclusion, we identified in this study those cancer cell lines with low level of expression and structural alteration of *PARP-1 gene*. A low level of *PARP-1* expression in human cancer could have a substantial impact on cancer cell growth, differentiation and cancer development by affecting genomic instability, as well as on the response of tumors to chemo- and radiotherapy.

## References

[1] Sugimura T. (1973) Prog. Nucleic Acid Res. Mol. Biol. 13, 127–151. 4349570 10.1016/s0079-6603(08)60102-6

[2] Bürkle A. Schreiber V. Dantzer F. Oliver F. J. Niedergang C.. (2000) In From DNA Damage And Stress Signaling to Cell Death PolyADP-Ribosylation Reactions (eds. de Murcia G. Shall S.). Oxford University Press, New York, pp. 80–124.

[3] Masutani M. Nakagama H. Sugimura T. (2003) Genes Chromosomes Cancer 38, 339–348. 14566854 10.1002/gcc.10250

[4] Rolli V. Ruf A. Augustin A. Schulz G. E. Ménissier-de Murcia J. de Murcia G. (2000) In From DNA Damage And Stress Signaling to Cell Death PolyADP-Ribosylation Reactions (eds. de Murcia G. Shall S.). Oxford University Press, New York, pp. 35–79.

[5] Shibata A. Kamada N. Masumura K. Nohmi T. Kobayashi S. Teraoka H. Nakagama H. Sugimura T. Suzuki H. Masutani M. (2003) Proceedings 62nd Annual Meeting of the Japanese Cancer Association, 94 (Suppl.), p. 251.

[6] Tsutsumi M. Masutani M. Nozaki T. Kusuoka O. Tsujiuchi T. Nakagama H. Suzuki H. Konishi Y. Sugimura T. (2001) Carcinog. 22, 1–3. 10.1093/carcin/22.1.1 11159733

[7] Yamamoto H. Okamoto H. (1980) Biochem. Biophys. Res. Commun. 95, 474–481. 6251809 10.1016/0006-291x(80)90762-7

[8] Yu S. W. Wang H. Poitras M. F. Coombs C. Bowers W. J. Federoff H. J. Poirier G. G. Dawson T. M. Dawson V. L. (2002) Science 297, 259–263. 12114629 10.1126/science.1072221

[9] Prasad S. C. Thraves P. J. Bhatia K. G. Smulson M. E. Dritschilo A. (1990) Cancer Res. 50, 38–43. 2104538

[10] Bhatia K. Huppi K. Cherney B. Raffeld M. Smulson M. Magrath I. (1990) Curr. Top. Microbiol. Immunol. 166, 347–357. 1981498 10.1007/978-3-642-75889-8_43

[11] Bhatia K. G. Cherney B. W. Huppi K. Magrath I. T. Cossman J. (1990) Cancer Res. 50, 5406–5413. 2117481

[12] Lyn D. Cherney B. W. Lalande M. Berenson J. R. Lichtenstein A. Lupold S. Bhatia K. G. Smulson M. (1993) Am. J. Hum. Genet. 52, 124–134. 8434580 PMC1682136

[13] Bieche I. de Murcia G. Lidereau R. (1996) Clin. Cancer Res. 2, 1163–1167. 9816283

[14] de Murcia J. M. Niedergang C. Trucco C. Ricoul M. Dutrillaux B. Mark M. Oliver F. J. Masson M. Dierich A. LeMeur M. Walztinger C. Chambon P. de Murcia G. (1997) Proc. Natl. Acad. Sci. USA 94, 7303–7307. 9207086 10.1073/pnas.94.14.7303 PMC23816

[15] Masutani M. Nozaki T. Nishiyama E. Shimokawa T. Tachi Y. Suzuki H. Nakagama H. Wakabayashi K. Sugimura T. (1999) Mol. Cell Biochem. 193, 149–152. 10331651

[16] Wang Z. Q. Stingl L. Morrison C. Jantsch M. Los M. Schulze-Osthoff K. Wagner E. F. (1997) Genes Dev. 11, 2347–2358. 9308963 10.1101/gad.11.18.2347 PMC316515

[17] Igaki H. Sasaki H. Kishi T. Sakamoto H. Tachimori Y. Kato H. Watanabe H. Sugimura T. Terada M. (1994) Biochem. Biophys. Res. Commun. 203, 1090–1095. 8093026 10.1006/bbrc.1994.2294

[18] Kohno T. Takakura S. Yamada T. Okamoto A. Tanaka T. Yokota J. (1999) Cancer Res. 59, 4170–4174. 10485448

[19] Gotoh M. Nakajima T. Yokota J. Tsunokawa Y. Terada M. Shimoyama Y. Teshima S. Hirohashi S. Shimosato Y. (1991) Jpn. J. Cancer Res. 82, 1252–1257. 1721614 10.1111/j.1349-7006.1991.tb01789.x PMC5918331

[20] Kishi T. Sasaki H. Akiyama N. Ishizuka T. Sakamoto H. Aizawa S. Sugimura T. Terada M. (1997) Biochem. Biophys. Res. Commun. 232, 5–9. 9125150 10.1006/bbrc.1997.6218

[21] Kodera T. Kohno T. Takakura S. Morishita K. Hamaguchi H. Hayashi Y. Sasaki T. Yokota J. (1999) Genes Chromosomes Cancer 26, 267–269. 10502327 10.1002/(sici)1098-2264(199911)26:3<267::aid-gcc13>3.0.co;2-v

[22] Shiraishi M. Noguchi M. Shimosato Y. Sekiya T. (1989) Cancer Res. 49, 6474–6479. 2573414

[23] Uchida K. Morita T. Sato T. Ogura T. Yamashita R. Noguchi S. Suzuki H. Nyunoya H. Miwa M. Sugimura T. (1987) Biochem. Biophys. Res. Commun. 148, 617–622. 3120710 10.1016/0006-291x(87)90921-1

[24] Masutani M. Nozaki T. Hitomi Y. Ikejima M. Nagasaki K. de Prati A. C. Kurata S. Natori S. Sugimura T. Esumi H. (1994) Eur. J. Biochem. 220, 607–614. 8125121 10.1111/j.1432-1033.1994.tb18662.x

[25] Chen T. R. Drabkowski D. Hay R. J. Macy M. Peterson W. Jr (1987) Cancer Genet. Cytogenet. 27, 125–134. 3472642 10.1016/0165-4608(87)90267-6

[26] Vilien M. Wolf H. (1978) J. Urol. 119, 338–342. 273706 10.1016/s0022-5347(17)57484-4

[27] Minowada J. Onuma T. Moore G. E. (1972) J. Natl. Cancer Inst. 49, 891–895. 4567231

[28] Foley G. E. Lazarus H. Farber S. Uzman B. G. Boone B. A. McCarthy R. E. (1965) Cancer 18, 522–529. 14278051 10.1002/1097-0142(196504)18:4<522::aid-cncr2820180418>3.0.co;2-j

[29] Motoyama T. Hojo H. Watanabe H. (1986) Acta Pathol. Jpn. 36, 65–83. 3962675 10.1111/j.1440-1827.1986.tb01461.x

[30] von Kobbe C. Harrigan J. A. May A. Opresko P. L. Dawut L. Cheng W. H. Bohr V. A. (2003) Mol. Cell Biol. 23, 8601–8613. 14612404 10.1128/MCB.23.23.8601-8613.2003 PMC262662

